# Supplementary material for: Non-coding Class Switch Recombination-Related Transcription in Human Normal and Pathological Immune Responses
Source: Front Immunol. 2018 Nov 21;9:2679. doi: 10.3389/fimmu.2018.02679 (PMC6260145; doi:10.3389/fimmu.2018.02679)
Supplement: Supplementary file 1 [file Data_Sheet_1.PDF]

Supplementary file 1

Non-coding Class Switch Recombination-related transcription in human normal and pathological immune responses

Helena Kuri-Magaña[1,2]; Leonardo Collado-Torres[3,4]; Andrew E. Jaffe[3,4,5,6]; Humberto Valdovinos-Torres[1]; Marbella Ovilla-Muñoz[1]; Juan M Téllez-Sosa[1]; Laura C Bonifaz Alfonzo[7]; Jesús Martínez-Barnetche[1*]

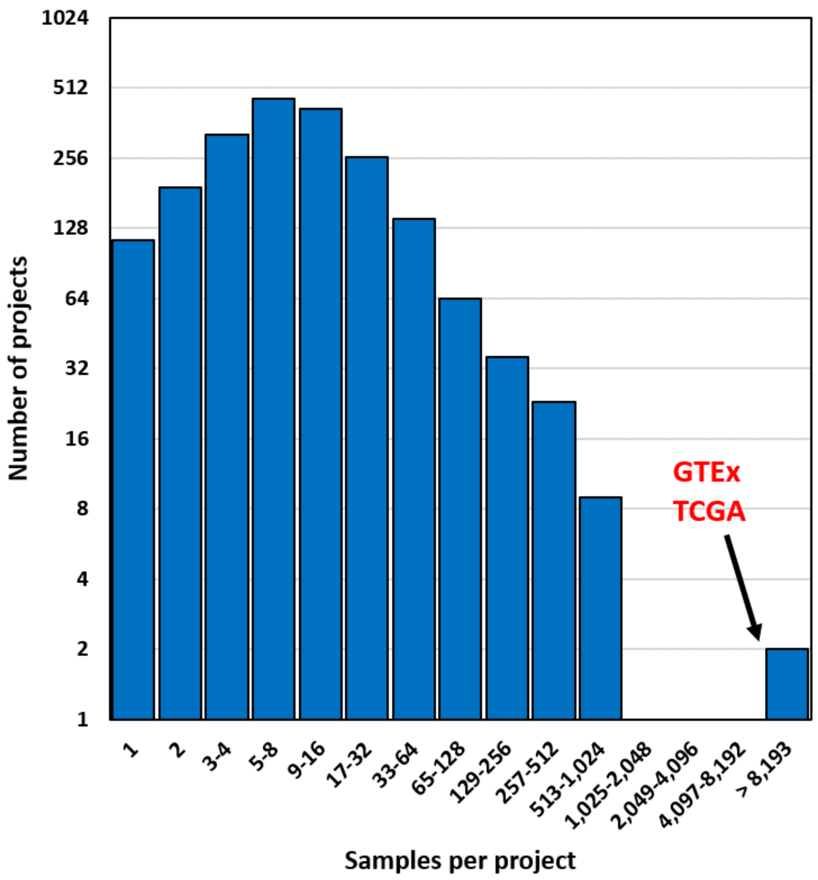

Figure S1: The whole *recount2* dataset was classified according to the number of samples per project (X-*axis*) and the number of projects (*Y-axis*). The number of projects analyzed with *recount2* was 2,036 with of 70,603 samples (Median = 8 samples per project; Mean = 32 samples per project). TCGA and GTEx are two projects that have 11,284 and 9,661 samples, respectively, comprising 29.6% of all samples.

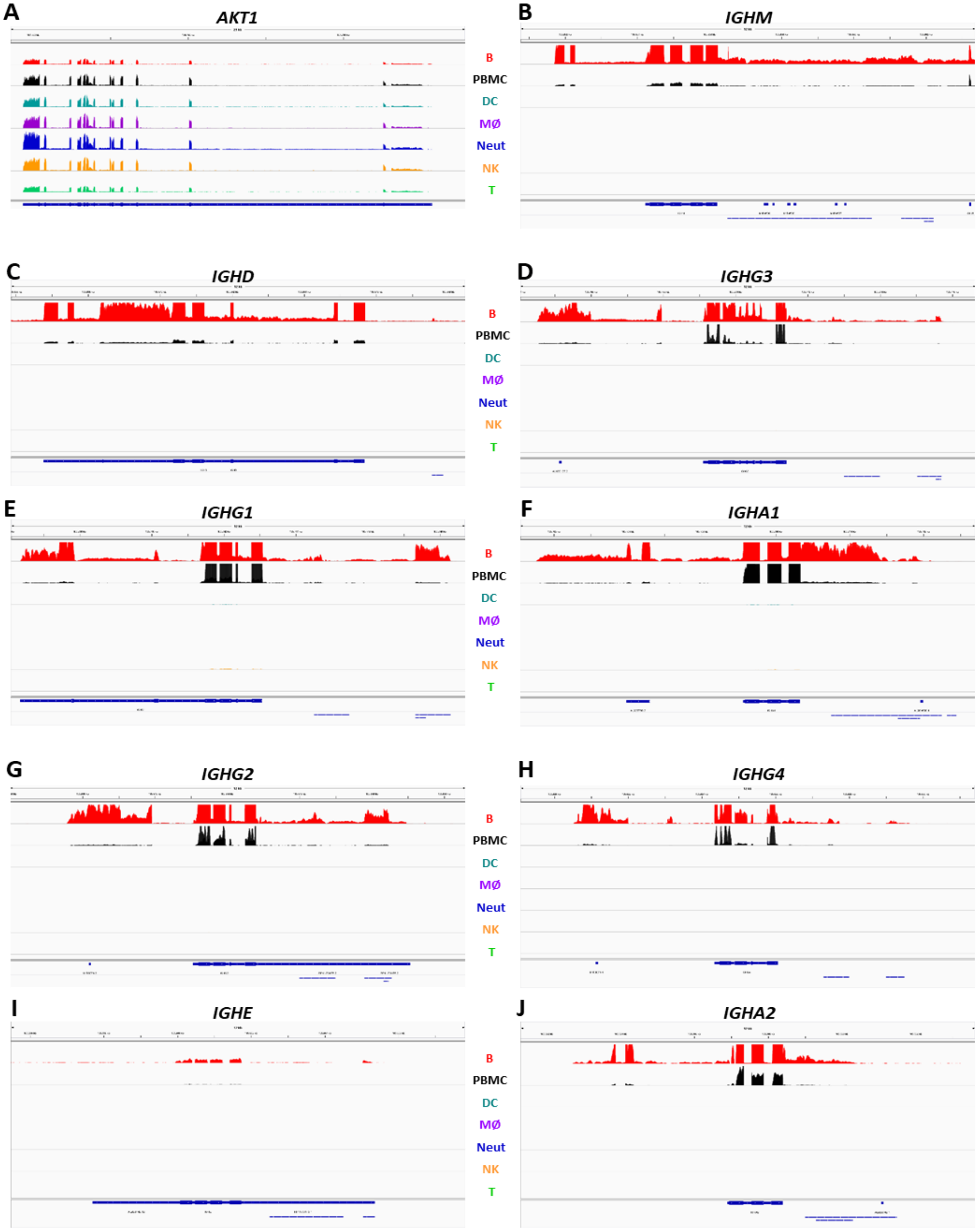

Figure S2: CSRnc transcription is B cells-specific. Coverage graphs of the *AKT1* (**A**) and IGH locus (**B-J**) showing transcriptional activity in isolated hematopoietic-derived differentiated cells from project SRP051688 (Hoek et al., 2015). B cells (B, red track), total PBMC's (PMBC, black track), myeloid dendritic cells (DC, sea green track), monocytes (MØ, purple track), neutrophils (Neut, blue track), Natural killer cells (NK, orange track) and T cells (T, green track).

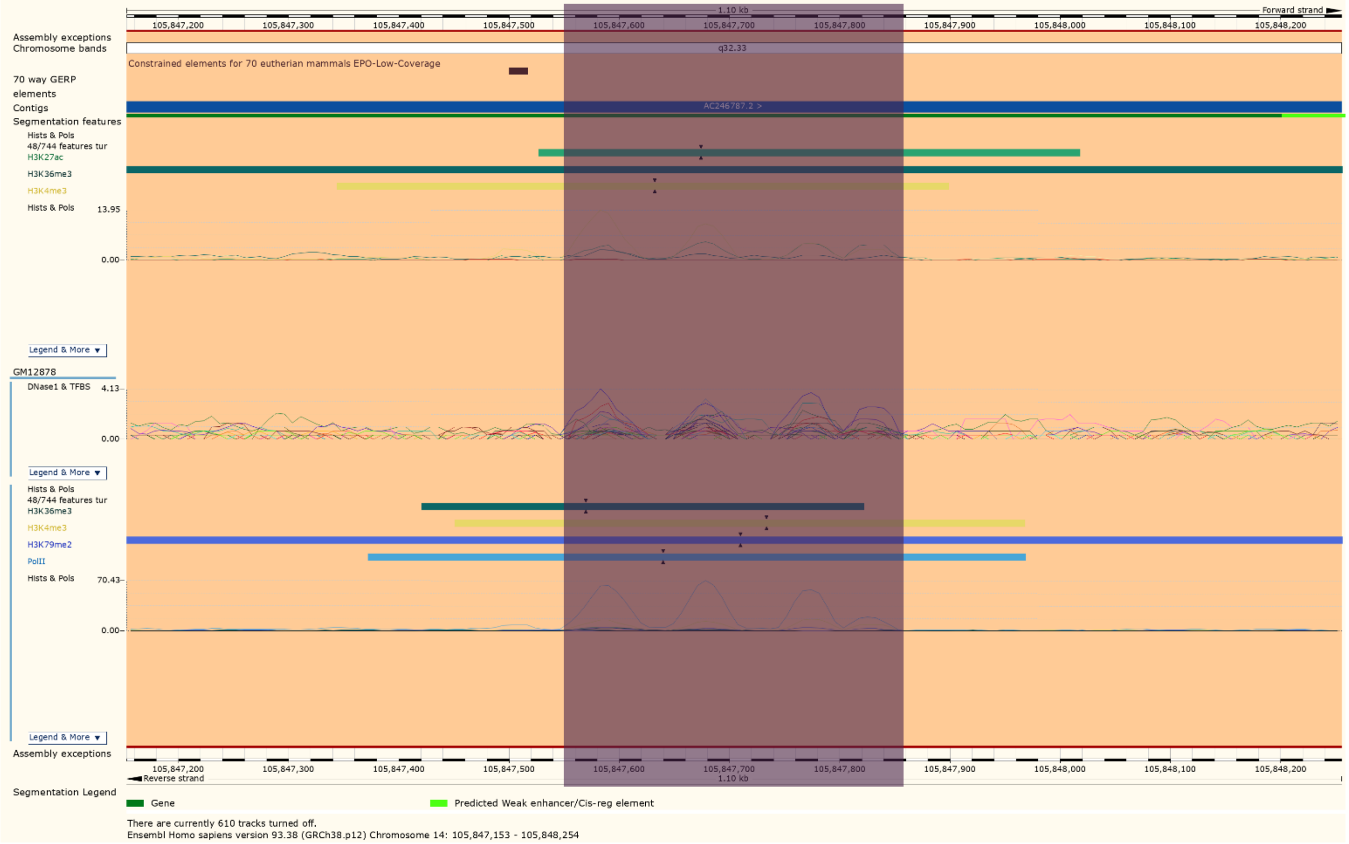

Figure S3: A novel transcribed region in the *IGHM-IGHD* intron locus (I_δ) displays epigenetic marks associated with active transcription in B cells. The ENSEMBL Genome Browser was used to search for epigenetic marks in the identified I_H of the IgH locus in human chromosome 14 (Zerbino et al., 2018). The upper track corresponds to the Roadmap Epigenomics project annotation for peripheral blood B cells (Roadmap Epigenomics et al., 2015). Signals of H3K36me3 (*dark green* trace) and H3K4me3 (*ochre* trace) and H3K27ac (*green* trace), all marks of active transcription overlap with the I_δ non-coding exon (*shaded rectangle*). The lower track corresponds to ENCODE project data on EBV-transformed lymphoblastoid cell line GM12878 (Consortium, 2012). In addition to H3K36me3 (*dark green* trace) and H3K4me3 (*ochre* trace) enrichment, H3K79me2 (*dark blue*) and PolII (*sky blue*) enrichment was identified.

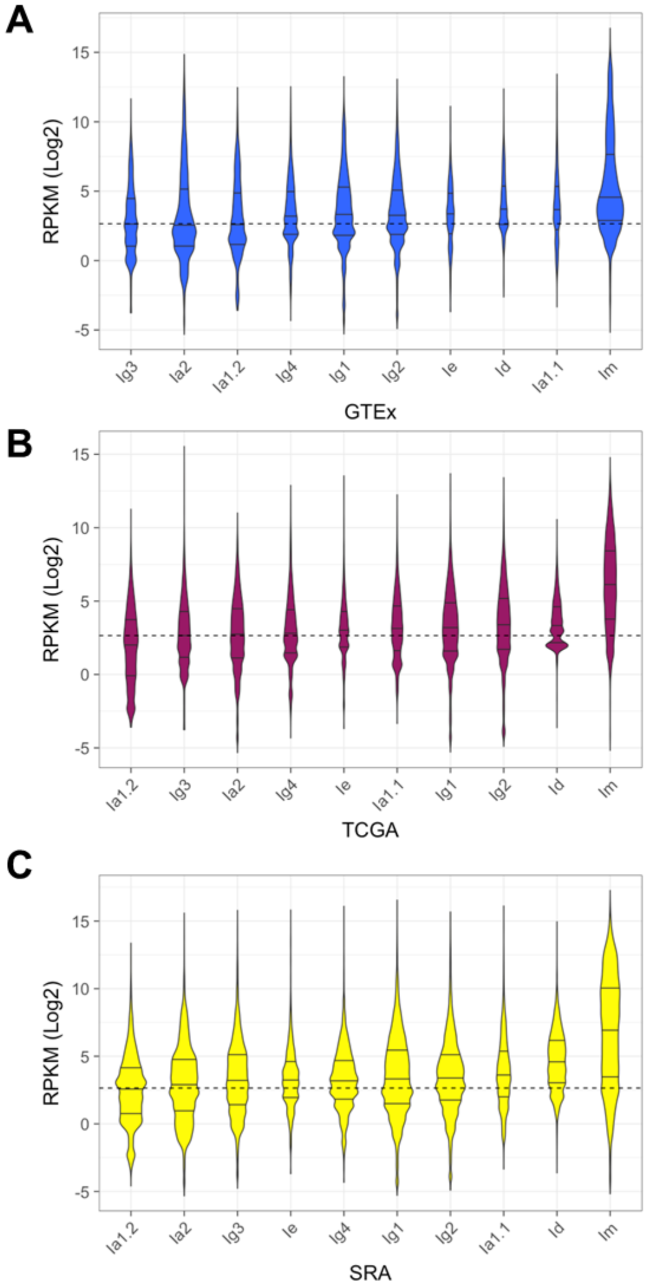

Figure S4: CSRnc transcription according to IH and project dataset. Violin plots of $\log_2$ RPKM distribution per CSRnc transcript (I_H) for (**A**) GTEx, (**B**) TCGA and (**C**) SRA datasets. The area of the violin is scaled to the amount of samples and violins are ordered according to the median $\log_2$ RPKM. Iμ has the highest transcription levels and is the most widely expressed. A sharp decrease in the transcription of remaining IH transcripts followed. Unexpectedly, Iδ and Iε transcription was relatively high, although its transcription was restricted to a small proportion of samples. Contrastingly, Iγ3 and Iα2 transcription levels were the lowest in all datasets and were transcribed in a relatively high proportion of samples. This data indicates that Iμ expression is higher and more widespread (i.e. expressed in many samples and projects of various origins), whereas other I_H transcription levels were usually lower and their transcription is more restricted.

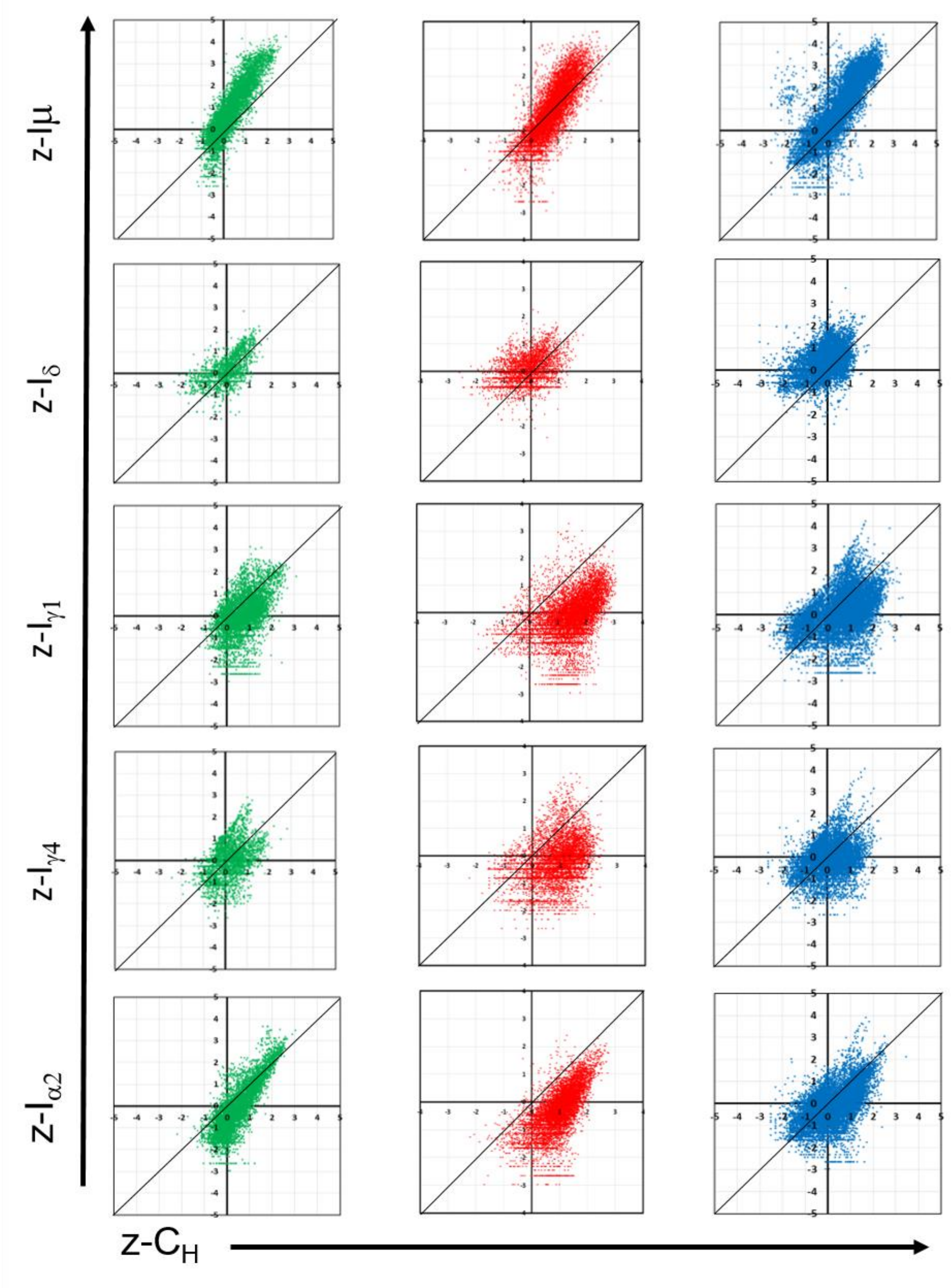

Figure S5: Correlation between CSRnc I_H transcription and coding C_H transcription. As for CSRnc I_H transcripts, the corresponding coding C_H log_2RPKM were transformed to Z-scores to make comparisons of their respective relative expression in two dimensional (C_H Z-score, *x* axis; I_H Z-score, *y* axis). Representative plots for I/C_μ, I/C_δ, $I/C_{\gamma1}$, $I/C_{\gamma4}$ and $I/C_{\alpha2}$ are shown for GTEx (*green*), TCGA (*red*) and SRA (*blue*). The black diagonal is shown to emphasize deviations from orthogonal plane. Samples above the diagonal indicate higher relative CSRnc transcription that it's corresponding coding C_H counterpart.

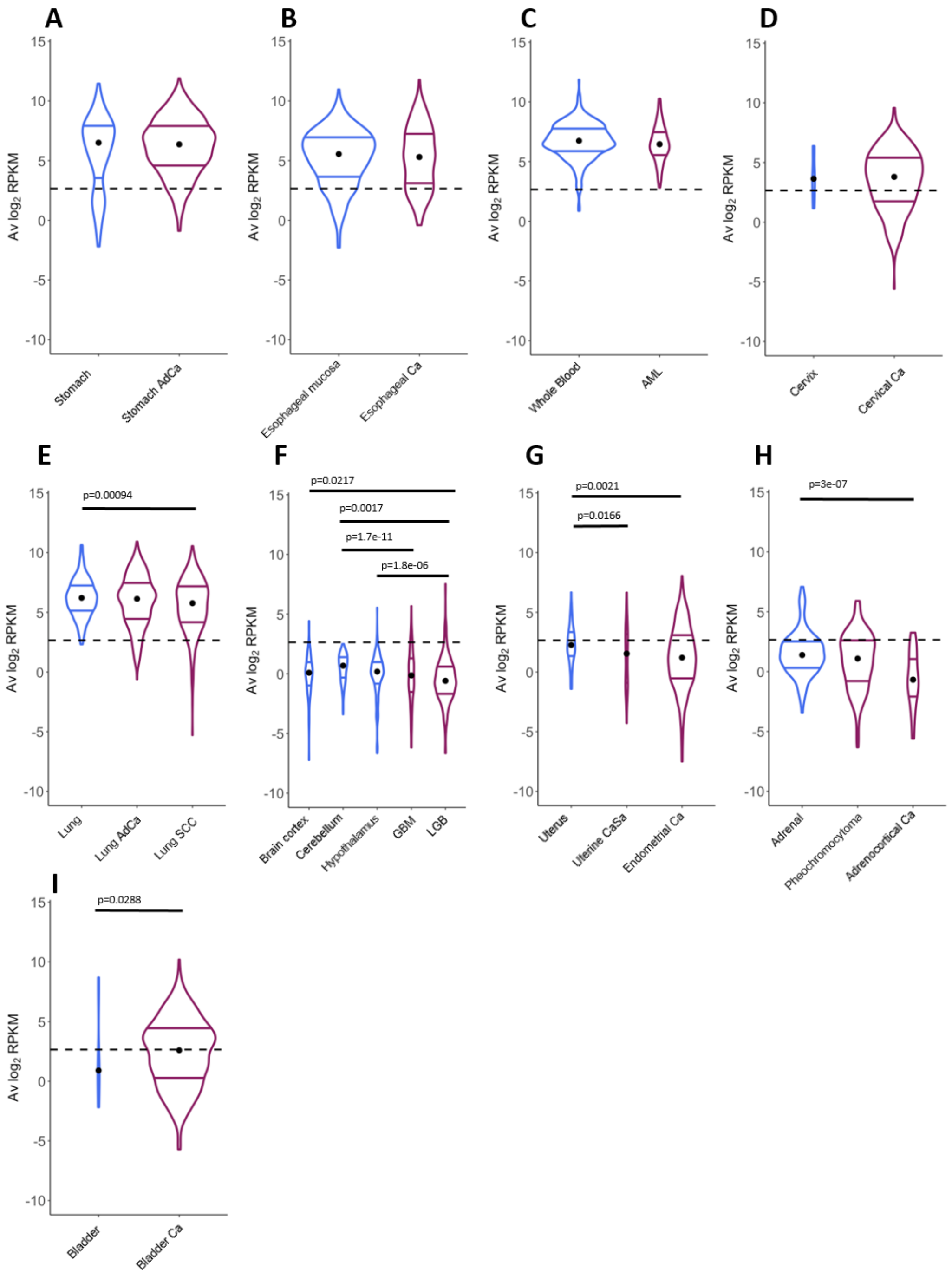

Figure S6: Comparison of CSRnc transcription in healthy tissue and its tumor counterpart. Violin plots of the average log_2RPKM distribution in healthy tissue (*blue*) in comparison with its cancer tissue counterpart (*purple*). Violin area corresponds to sample count. Median (*black dot*) and quartiles are shown for each violin. Dashed black line marks the mean average log_2RPKM (2.65). No differences in CSRnc transcription in tumors was observed when compared to its healthy counterpart in **A-D**. No differences were detected in healthy lung and lung adenocarcinoma, however CSRnc transcription was lower in lung squamous cell carcinoma (**E**). Lower CSRnc transcription was also noted in central nervous system tumors (**F**), uterine carcinosarcoma and endometrial carcinoma (**G**) and adrenocortical carcinoma, but not pheochromocytoma (**H**). Increased CSRnc transcription was detected in bladder cancer (**I**). The conducted statistical test were Wilcoxon rank sum test with continuity correction for two-sample comparisons, and Kruskal-Wallis test with *post hoc* Dunn's test correction for multiple comparisons.
