# Supplementary material for: Non-coding Class Switch Recombination-Related Transcription in Human Normal and Pathological Immune Responses
Source: Front Immunol. 2018 Nov 21;9:2679. doi: 10.3389/fimmu.2018.02679 (PMC6260145; doi:10.3389/fimmu.2018.02679)
Supplement: Supplementary file 2 [file Data_Sheet_2.PDF]

Supplementary file 2

Non-coding Class Switch Recombination-related transcription in human normal and pathological immune responses

Helena Kuri-Magaña[1,2]; Leonardo Collado-Torres[3,4]; Andrew E. Jaffe[3,4,5,6]; Humberto Valdovinos-Torres[1]; Marbella Ovilla-Muñoz[1]; Juan M Téllez-Sosa[1]; Laura C Bonifaz Alfonzo[7]; Jesús Martínez-Barnetche[1*]

Table S1: Selected SRA projects used to map I_H boundaries

SRA Study	Samples	Cell type	Study	Ref.
**SRP020491**				
	SRR805725	Circulating B cells (pre-vaccine)	Changes in gene expression profiles of circulating B cells after influenza vaccination in healthy human subjects	Henn *et al*., 2013
	SRR805736	Circulating B cells (pre-vaccine)		
	SRR805747	Circulating B cells (pre-vaccine)		
	SRR805758	Circulating B cells (pre-vaccine)		
	SRR805769	Circulating B cells (pre-vaccine)		
**SRP021509**				
	SRR834979	Tonsillar naive B cells	EZH2 is required for germinal center formation and somatic EZH2 mutations promote lymphoid transformation	Béguelin *et al*., 2013
	SRR834980	Tonsillar naive B cells		
	SRR834981	Tonsillar naive B cells		
	SRR834982	Tonsillar naive B cells		
	SRR834983	Tonsillar Germinal Center B cells		
	SRR834984	Tonsillar Germinal Center B cells		
	SRR834985	Tonsillar Germinal Center B cells		
	SRR834986	Tonsillar Germinal Center B cells		
**SRP033335**				
	SRR1038580	Control CD19+ B cells	Genome-wide expression profiling of B Lymphocytes reveals IL4R increase in allergic asthma	Pascual *et al*., 2014
	SRR1038581	Control CD19+ B cells		
	SRR1038582	Control CD19+ B cells		
**SRP045500**				
	SRR1550988	Healthy control B cells	Next generation sequencing of human immune cell subsets across diseases	Linsley *et al*., 2014
	SRR1551042	Healthy control B cells		
	SRR1551049	Healthy control B cells		
	SRR1551070	Healthy control B cells		
**SRP048820**				
	SRR1609989	Centrocyte	Enhancer Sequence Variants and Transcription Factor Deregulation Synergize to Construct Pathogenic Regulatory Circuits in B Cell Lymphoma	Koues *et al*., 2014
	SRR1609990	Centrocyte		
	SRR1609991	PB Activated B cells		
	SRR1609992	PB Activated B cells		

**SRP051688**				
	SRR1740034	Primary B cells (pre-vaccine)	A Cell-based Systems Biology Assessment of Human Blood to Monitor Immune Responses After Influenza Vaccination	Hoek *et al*., 2013
	SRR1740062	Primary B cells (pre-vaccine)		
**SRP055390**				
	SRR1812749	Normal CD19+/CD27+ memory B-cells	Transcriptome analysis in chronic lymphocytic leukemia cells using RNA sequencing (RNA-seq)	Kushwaha *et al*., 2016
	SRR1812750	Normal CD19+/ IgD+ naive B-cells		
	SRR1812751	Normal CD19+ B-cells		
	SRR1812752	Normal CD19+ B-cells		
	SRR1812753	Normal CD19+ B-cells		
**SRP060715**				
	SRR2097512	Purified normal B cells	Gene expression and splicing alterations analyzed by high throughput RNA sequencing of chronic lymphocytic leukemia specimens	Unpublished
	SRR2097513	Purified normal B cells		
	SRR2097514	Purified normal B cells		
	SRR2097515	Purified normal B cells		
	SRR2097516	Purified normal B cells		

*Data shown as it is displayed for each of the projects in the SRA

Table S2. CSRnc transcription in cancer cell lines

	Samples with High_expression	Samples with low/no expression	p_val	fdr	
acute lymphocytic leukemia	6	111	0.4732	0.6310	
acute monocytic leukemia	12	142	0.7540	0.9280	
**acute myeloid leukemia**	**2**	**185**	**0.0002**	**0.0003**	*
prostate cancer	13	319	0.0129	0.0206	
pancreatic cancer	0	58	0.0207	0.0301	
**colorectal cancer**	**9**	**580**	**0.0000**	**0.0000**	*
**breast cancer**	**32**	**1417**	**0.0000**	**0.0000**	*
**lung cancer**	**0**	**462**	**0.0000**	**0.0000**	*
gastric cancer	3	46	1.0000	1.0000	
adrenal cancer	0	3	1.0000	1.0000	
**B cell lymphoma**	**77**	**38**	**0.0000**	**0.0000**	*
**astrocytoma**	**1**	**534**	**0.0000**	**0.0000**	*
**cervical cancer**	**7**	**1613**	**0.0000**	**0.0000**	*
**chronic myeloid leukemia**	**8**	**522**	**0.0000**	**0.0000**	*
**osteosarcoma**	**3**	**159**	**0.0034**	**0.0060**	*
cholangiosarcoma	0	7	1.0000	1.0000	

Exact Fisher's test. **Enriched.** **Depleted**

** Benjamini-Hochberg post hoc correction for multiple comparisons.

Table S3. Differential expression analysis in IH and IGH

				coding									p-value *						non coding			
**Project_ID**	**Description**	**Comparisson**	**Sample type**	**Cm**	**Cd**	**Cg3**	**Cg1**	**Ca1**	**Cg2**	**Cg4**	**Ce**	**Ca2**	**Im**	**Id**	**Ig3**	**Ig1**	**Ia1.1**	**Ia1.2**	**Ig2**	**Ig4**	**Ie**	**Ia2**
SRP032775	Malaria	Post-infection vs Pre-infection	Whole blood	1.00	**0.01**	1.00	1.00	1.00	1.00	1.00	1.00	1.00	0.92	0.14	0.15	**0.05**	1.00	1.00	0.16	1.00	1.00	1.00
SRP033696	Influenza	H7N9 infection vs healthy controls	Whole blood	**0.00**	1.00	0.23	0.38	**0.00**	**0.00**	**0.04**	1.00	0.05	1.00	1.00	0.49	**0.02**	1.00	**0.00**	0.47	1.00	1.00	1.00
SRP059039	Pediatric diarrhea	*Shigella* vs Control	Whole blood	0.98	**0.03**	1.00	1.00	1.00	1.00	1.00	1.00	1.00	**0.03**	0.74	0.13	**0.01**	1.00	0.89	0.51	**0.03**	1.00	1.00
SRP059039	Pediatric diarrhea	*Salmonella* vs Control	Whole blood	1.00	1.00	1.00	1.00	1.00	0.55	1.00	1.00	1.00	1.00	1.00	**0.03**	0.08	1.00	1.00	**0.05**	0.25	1.00	1.00
SRP059039	Pediatric diarrhea	Rotavirus vs Control	Whole blood	1.00	0.53	1.00	1.00	1.00	0.42	1.00	1.00	1.00	1.00	1.00	1.00	0.43	1.00	1.00	0.05	0.36	1.00	1.00
SRP059172	Brucellosis/Leishmaniasis infection	*Brucella* vs Control	Whole blood	1.00	1.00	1.00	0.85	1.00	1.00	1.00	1.00	1.00	0.42	**0.00**	1.00	1.00	1.00	0.72	0.61	1.00	0.35	1.00
SRP059172	Brucellosis/Leishmaniasis infection	*Leishmania* vs Control	Whole blood	1.00	**0.00**	0.07	**0.01**	0.76	0.57	0.05	1.00	0.34	1.00	1.00	0.20	0.47	1.00	1.00	**0.01**	1.00	0.48	1.00
SRP042228	Pediatric Crohn	Crohn's disease (iCD) vs Not-IBD	Ileal biopsy	**0.00**	1.00	**0.01**	**0.01**	**0.00**	**0.00**	**0.00**	1.00	**0.00**	**0.00**	**0.03**	1.00	1.00	**0.01**	0.54	0.91	1.00	1.00	**0.02**
SRP042228	Pediatric Crohn	Crohn's disease (cCD) vs Not-IBD	Ileal biopsy	0.32	1.00	0.14	0.14	0.07	**0.02**	**0.02**	1.00	**0.04**	0.14	0.28	1.00	1.00	1.00	1.00	1.00	1.00	1.00	1.00
SRP042228	Pediatric Crohn	Ulcerative colitis vs Not-IBD	Ileal biopsy	1.00	1.00	1.00	1.00	1.00	1.00	1.00	1.00	1.00	1.00	1.00	1.00	1.00	1.00	1.00	1.00	1.00	1.00	0.86
SRP051688	Influenza	Post-immune(day 7) vs preimmune	B cells	1.00	1.00	1.00	0.07	1.00	1.00	1.00	1.00	0.89	1.00	1.00	1.00	1.00	1.00	1.00	1.00	1.00	1.00	1.00
SRP020491	Influenza	Post-immune(day 7) vs preimmune	PBMC	1.00	1.00	1.00	0.27	1.00	1.00	0.95	1.00	1.00	1.00	1.00	1.00	1.00	1.00	1.00	1.00	1.00	1.00	1.00
SRP062966	Systemic Lupus Erythematosus (SLE)	SLE patients vs healthy control	Whole blood	1.00	1.00	0.99	0.56	0.19	1.00	1.00	1.00	0.31	0.93	0.82	1.00	1.00	1.00	1.00	0.39	1.00	0.48	0.77

* p- value adjusted by Bonferroni. Values in green represent a significant decrease of expression compared to control. Values in red represent a significant increase of expression compared to control.
